# Clinicopathological Characteristics and Treatment Strategies of Triple-Negative Breast Cancer Patients With a Survival Longer than 5 Years

**DOI:** 10.3389/fonc.2020.617593

**Published:** 2021-02-01

**Authors:** Ning Xie, Ying Xu, Ying Zhong, Junwei Li, Herui Yao, Tao Qin

**Affiliations:** ^1^ Department of Medical Oncology, Sun Yat-sen Memorial Hospital, Sun Yat-sen University, Guangzhou, China; ^2^ Guangdong Provincial Key Laboratory of Malignant Tumor Epigenetics and Gene Regulation, Sun Yat-sen Memorial Hospital, Sun Yat-sen University, Guangzhou, China; ^3^ Breast Tumor Center, Sun Yat-sen Memorial Hospital, Sun Yat-sen University, Guangzhou, China

**Keywords:** triple negative breast cancer, clinicopathological characteristics, overall survival, breast cancer cause-specific survival, SEER database

## Abstract

**Purpose:**

Triple-negative breast cancer (TNBC) is characterized by high malignancy and a poor prognosis. Patients with TNBC who survive longer than 5 years represent a unique portion of the population. This study aimed to analyze the clinicopathological features, explore prognostic factors, and evaluate treatment options for these patients.

**Methods:**

A total of 24,943 TNBC patients were enrolled from the national Surveillance, Epidemiology, and End Results (SEER) database between January 2010 and December 2016. The patients were divided into three groups: group 1, survival time <3 years; group 2, 3–5 years; and group 3, survival time ≥5 years. The overall survival (OS) and breast cancer cause-specific survival (BCSS) were primarily assessed in this study. A propensity score analysis was used to avoid bias caused by the data selection criteria. We used a Cox hazard ratio analysis to determine prognostic factors, which were selected as nomogram parameters to develop a model for predicting patient survival.

**Results:**

Patients who survived longer than 5 years were more likely to be younger than 55 years, Caucasian, and exhibit a lower AJCC stage, N stage, distant metastasis, lymph node (LN) involvement, and tumor size than those with a shorter survival time (*p* < 0.05). The multivariable Cox regression analysis showed that age, race, tumor size, LN status, and chemotherapy were independent prognostic factors. Subgroup analyses for patients with tumors ≤20 mm displayed a superior OS and BCSS for breast-conserving surgery (BCS) not treated with a mastectomy. BCS provided at least an equivalent prognosis to a mastectomy in patients with tumors larger than 20 mm. A nomogram with a C-index of 0.776 (95% confidence interval: 0.767–0.785) was developed to predict the 3- and 5-year survival probability for the patients with TNBC.

**Conclusion:**

A localized surgical approach may represent a superior choice for TNBC patients with a survival time longer than 5 years. Our study indicated that age, race, tumor size, LN status, and chemotherapy were independent prognostic factors. A prognostic nomogram directly quantified patient risk and was better able to predict long-term survival in TNBC patients.

## Introduction

Triple-negative breast cancer (TNBC) is a phenotypic subtype defined by a lack of hormonal (estrogen and progesterone) receptors and human epidermal growth factor receptor 2 (HER2) expression that accounts for 10% to 20% of all breast cancers (BC) (1–3). Moreover, TNBC is characterized by more aggressive and easier distant metastases (4–6). TNBC also has characteristics of significant heterogeneity and a poor prognosis. Due to the lack of a standard chemotherapy regimen and surgical treatment for TNBC, it has become an area of keen interest in the medical field. Patients with TNBC who survived longer than 5 years represent a unique portion of the population. In addition, there is a lack of supporting evidence for any specific treatment guidelines for these patients. Given the lack of available accurate information concerning the relevant prognostic factors for patients with TNBC who survived longer than 5 years, the best surgical approach and the most suitable treatment strategy remains unavailable. Furthermore, the outcomes between breast-conserving surgery (BCS) and mastectomy remain controversial. When selecting a strategy, life expectancy, potential benefits of treatment, the patient’s goals for treatment, and potential risks associated with treatment should be considered.

The aim of this study was to investigate the clinicopathological characteristics of TNBC patients who survived longer than 5 years, and identify the underlying prognostic factors likely to be the most useful to clinicians making treatment decisions. To this end, we obtained the most recent population-based data by utilizing the Surveillance, Epidemiology, and End Results (SEER) database. We also built a nomogram-derived overall survival (OS) estimation system that could be used to provide more accurate predictions of the 3- and 5-year survival probability for patients with TNBC. The large size of the database and the variable patient population allows for the investigation of clinical predictors of TNBC and a detailed description of its behavior.

## Materials and Methods

### Patients

All patients were enrolled from the SEER database from 2010 to 2016, since the HER-2 status was not available until after 2010. The SEER database was used to collect and report the patients’ clinicopathological features and survival data, including authoritative information about cancer incidence and survival data from 18 population-based cancer registries, covering approximately one-third of the U.S. population. The following inclusion criteria were used: the diagnosis was confirmed by positive histology and the molecular subtype of the breast was confirmed to be triple negative by pathological analysis. Patients without a histological diagnosis and survival data were excluded. In our study, a signed SEER research data agreement form was provided to the SEER program to obtain approval to access and analyze the SEER database. Since the SEER database is available to the public, use of the data does not require ethical approval. To further analyze the clinicopathological characteristics of the patients with TNBC, they were divided into three groups: group 1, survival time <3 years; group 2, 3–5 years; and group 3, survival time ≥5 years. For nomogram construction and validation, all of the patients were randomly divided into training and validation cohorts at a 1:1 ratio.

### Outcome Measurement

The OS and breast cancer cause-specific survival (BCSS) were the main endpoints based on the data in the SEER database. OS was defined as the time from the date of diagnosis to the date of death due to any cause or the last follow-up. BCSS was defined as the time from the date of diagnosis to the date of death from BC. Survival time was defined as the duration from the initial diagnosis to death from any cause or to the last follow-up. Baseline characteristics were assessed to determine whether there were significant differences in the distribution of the study population. To evaluate the discriminative ability of the nomogram, a concordance index (C-index) and the receiver operating characteristic curve (ROC) were used and the area under the curve (AUC) was assessed. The calibration curve was used to compare the association between the actual outcomes and the predicted probabilities.

### Statistical Analysis

The clinicopathological characteristics were compared using Pearson chi-square and Fisher’s exact probability tests. Univariate and multivariate Cox proportional hazard models were applied to identify prognostic factors. OS and BCSS survival curves were calculated using the Kaplan Meier method, and significance among the different groups was assessed with a log-rank test. The nomogram was constructed to predict the survival probability based on independent significant variables. The C-indices were performed with the rcorrpcens in “Hmisc” package in R. To adjust the comparisons and avoid distortions from bias in retrospective trials, propensity score matching (PSM) was used with “MatchIt” packages. All statistical analyses were performed using SPSS statistical 25.0 (IBM Corporation, Armonk, NY, USA) and R 4.0.0 (R Development Core Team, R Foundation for Statistical Computing, Vienna, Austria) software. All statistical tests were two-sided, and a threshold of *p* < 0.05 was considered to be statistically significant for all the statistical tests.

## Results

### Baseline Characteristics of the Study Population

A total of 24,943 patients with TNBC from January 2010 to December 2016 were identified in the SEER database. The detailed clinicopathological characteristics are summarized in **Table 1**. Compared with group 1 and group 2, group 3 was more likely to be younger than 55 years (G3 vs. G1 and G2: 42.2% vs. 35.9% and 38.4%), had a higher proportion of Caucasians (75.5% vs. 69.5% and 71.4%) and first malignant primary indicator (83.9% vs. 79.1% and 80.3%); lower American Joint Committee on Cancer (AJCC) stage (III–IV, 11.0% vs. 23.0% and 12.8%), N stage (N0, 72.0% vs. 43.4% and 69.7%), distant metastasis (M1, 0.8% vs. 9.0% and 1.7%), lymph node (LN) involvement (negative, 69.6% vs. 51.0% and 65.0%), and smaller tumor size (≤20 mm, 50.8% vs. 27.9% and 48.3%). Concerning treatment options, patients in group 3 were more likely to receive BCS (52.2% vs. 42.2% and 50.0%), chemotherapy (73.0% vs. 69.3% and 71.0%), and radiotherapy (51.1% vs. 42.5% and 47.7%) compared to patients in groups 1 and 2 (*p* < 0.05). There was no significant difference in the tumor grade (III–IV, 75.9% vs. 76.4% and 75.7%) and laterality (Left, 51.1% vs. 51.0% and 52.1%) among the three groups.

**Table 1:** Baseline characteristics of patients with different survival times.

Characteristics	ST <3Y(*n* = 13470)	ST 3-5Y(*n* = 5807)	ST ≥5Y(*n* = 5666)	*p* value
Age				<0.001
<55 years	4,833 (35.9)	2,227 (38.4)	2,389 (42.2)	
≥55 years	8,637 (64.1)	3,580 (61.6)	3,277 (57.8)	
Race				<0.001
White	9,365 (69.5)	4,146 (71.4)	4,277 (75.5)	
Black	2,559 (19.0)	1,023 (17.6)	822 (14.5)	
Other	1,463 (10.9)	607 (10.5)	540 (9.5)	
Unknown	83 (0.6)	31 (0.5)	27 (0.5)	
Grade				<0.001
I	238 (1.8)	148 (2.5)	163 (2.9)	
II	2,257 (16.8)	1,042 (17.9)	1,027 (18.1)	
III	10,216 (75.8)	4,354 (75.0)	4,239 (74.8)	
IV	78 (0.6)	39 (0.7)	60 (1.1)	
Unknown	681 (5.1)	224 (3.9)	177 (3.1)	
Tumor size				<0.001
≤20 mm	3,760 (27.9)	2,802 (48.3)	2,878 (50.8)	
21–50 mm	4,281 (31.8)	2,408 (41.5)	2,254 (39.8)	
>50 mm	1,700 (12.6)	454 (7.8)	415 (7.3)	
Unknown	3,729 (27.7)	143 (2.5)	119 (2.1)	
Marital status				<0.001
Unmarried	2,456 (18.2)	978 (16.8)	888 (15.7)	
Married	10,277 (76.3)	4,519 (77.8)	4,480 (79.1)	
Unknown	737 (5.5)	310 (5.3)	298 (5.3)	
Laterality				<0.001
Right	6,551 (48.6)	2,775 (47.8)	2,768 (48.9)	
Left	6,874 (51.0)	3,026 (52.1)	2,895 (51.1)	
Others	45 (0.3)	6 (0.1)	3 (0.1)	
Stage				<0.001
0	0 (0)	1 (0)	1 (0)	
I	2,957 (22.0)	2,362 (40.7)	2,472 (43.6)	
II	3,980 (29.5)	2,584 (44.5)	2,471 (43.6)	
III	1,889 (14.0)	644 (11.1)	580 (10.2)	
IV	1,212 (9.0)	97 (1.7)	45 (0.8)	
Unknown	3,432 (25.5)	119 (2.0)	97 (1.7)	
T stage				<0.001
0	46 (0.3)	20 (0.3)	21 (0.4)	
T1	3,664 (27.2)	2,773 (47.8)	2,856 (50.4)	
T2	3,957 (29.4)	2,344 (40.4)	2,197 (38.8)	
T3	1,122 (8.3)	374 (6.4)	356 (6.3)	
T4	1,143 (8.5)	188 (3.2)	138 (2.4)	
Unknown	3,538 (26.3)	108 (1.9)	98 (1.7)	
N stage				<0.001
N0	5,845 (43.4)	4,048 (69.7)	4,081 (72.0)	
N1	2,725 (20.2)	1,264 (21.8)	1,186 (20.9)	
N2	731 (5.4)	276 (4.8)	248 (4.4)	
N3	770 (5.7)	156 (2.7)	119 (2.1)	
Unknown	3,399 (25.2)	63 (1.1)	32 (0.6)	
Metastasis				<0.001
M0	9,123 (67.7)	5,704 (98.2)	5,618 (99.2)	
M1	1,212 (9.0)	97 (1.7)	45 (0.8)	
Unknown	3,135 (23.3)	6 (0.1)	3 (0.1)	
LN Status				<0.001
Negative	6,869 (51.0)	3,777 (65.0)	3,942 (69.6)	
1–3 LN	2169 (16.1)	1,000 (17.2)	998 (17.6)	
>3 LN	1,259 (9.3)	342 (5.9)	299 (5.3)	
Unknown	3,173 (23.6)	688 (11.8)	427 (7.5)	
Negative	6,869 (51.0)	3,777 (65.0)	3,942 (69.6)	
1–3 LN	2,169 (16.1)	1,000 (17.2)	998 (17.6)	
>3 LN	1,259 (9.3)	342 (5.9)	299 (5.3)	
Unknown	3,173 (23.6)	688 (11.8)	427 (7.5)	
Surgery				<0.001
No surgery	2,166 (16.1)	247 (4.3)	149 (2.6)	
Breast-conserving surgery	5,688 (42.2)	2,902 (50.0)	2,956 (52.2)	
Mastectomy	5,584 (41.5)	2,650 (45.6)	2,556 (45.1)	
Unknown	32 (0.2)	8 (0.1)	5 (0.1)	
Radiation				<0.001
No	7,750 (57.5)	3,037 (52.3)	2,773 (48.9)	
Yes	5,720 (42.5)	2,770 (47.7)	2,893 (51.1)	
Chemotherapy				<0.001
No	4,131 (30.7)	1,685 (29.0)	1,531 (27.0)	
Yes	9,339 (69.3)	4,122 (71.0)	4,135 (73.0)	
Bone metastasis				<0.001
No	12,855 (95.4)	5,769 (99.3)	5,652 (99.8)	
Yes	615 (4.6)	38 (0.7)	14 (0.2)	
Brain metastasis				<0.001
No	13,293 (98.7)	5,805 (100)	5,665 (100)	
Yes	177 (1.3)	2 (0)	1 (0)	
Liver metastasis				<0.001
No	13,056 (96.9)	5,794 (99.8)	5,661 (99.9)	
Yes	414 (3.1)	13 (0.2)	5 (0.1)	
Lung metastasis				<0.001
No	12,885 (95.7)	5,782 (99.6)	5,655 (99.8)	
Yes	585 (4.3)	25 (0.4)	11 (0.2)	
Status				<0.001
Alive	9,161 (68.0)	4,907 (84.5)	5,474 (96.6)	
Dead	4,309 (32.0)	900 (15.5)	192 (3.4)	
First malignant primary indicator			<0.001
No	2,809 (20.9)	1,142 (19.7)	915 (16.1)	
Yes	10,661 (79.1)	4,665 (80.3)	4,751 (83.9)	
Sequence number				0.056
One primary only	9,743 (72.3)	4,139 (71.3)	4,152 (73.3)	
More primaries	3,727 (27.7)	1,668 (28.7)	1,514 (26.7)	

ST, survival time; Y, year; LN, lymph node.

Group 3 also had higher marriage rates (79.1% vs. 76.3%; *p* < 0.001), lower T stage (III–IV, 8.7% vs. 16.8%; *p* < 0.001) compared to group 1. In addition, we failed to identify a difference in the marriage rate (77.8% vs. 79.1%; *p* = 0.104), and T stage (III–IV, 9.7% vs. 8.7%; *p* = 0.076) between groups 2 and 3. Compared with group 1, the patients in group 2 were more likely to be younger than 55 years (38.4% vs. 35.9%; *p* = 0.001) and had a higher proportion of Caucasians (71.4% vs. 69.5%; *p* = 0.009), marriage rates (77.8% vs. 76.3%; *p* = 0.022), and smaller tumor size (≤2 cm, 48.3% vs. 27.9%; *p* < 0.001); lower AJCC stage (III–IV, 12.8% vs. 23.0%; *p* < 0.001), T stage (III–IV, 9.7% vs. 16.8%; *p* < 0.001), N stage (N0, 69.7% vs. 43.4%; *p* < 0.001), distant metastasis (M1, 1.7% vs. 9.0%; *p* < 0.001), and LN involvement (negative, 65.0% vs. 51.0%; *p* < 0.001). The patients in group 2 were also more likely to receive BCS (50.0% vs. 42.2%; *p* < 0.001), chemotherapy (71.0% vs. 69.3%; *p* = 0.022), and radiotherapy (47.7% vs. 42.5%; *p* < 0.001) compared to the patients in group 1. There was no significant difference in the grade (III–IV, 75.7% vs. 76.4%; *p* = 0.249), laterality (Left, 52.1% vs. 51.0%; *p* = 0.170), and first malignant primary indicator between the two groups (80.3% vs. 79.1%; *p* = 0.061). Subgroup analyses for stage IV patients showed that group 2 and group 3 had lower rate of bone (39.2% and 31.1% vs. 50.7%), brain (2.1% and 2.2% vs. 14.6%), liver (13.4% and 11.1% vs. 34.2%), and lung (25.8% and 24.4% vs. 48.3%) metastasis compared with that of group 1 (*p* < 0.05).

### Prognostic Factors and Treatment Outcomes for Patients With a Survival Time Longer Than 5 Years

**Figure 1:**
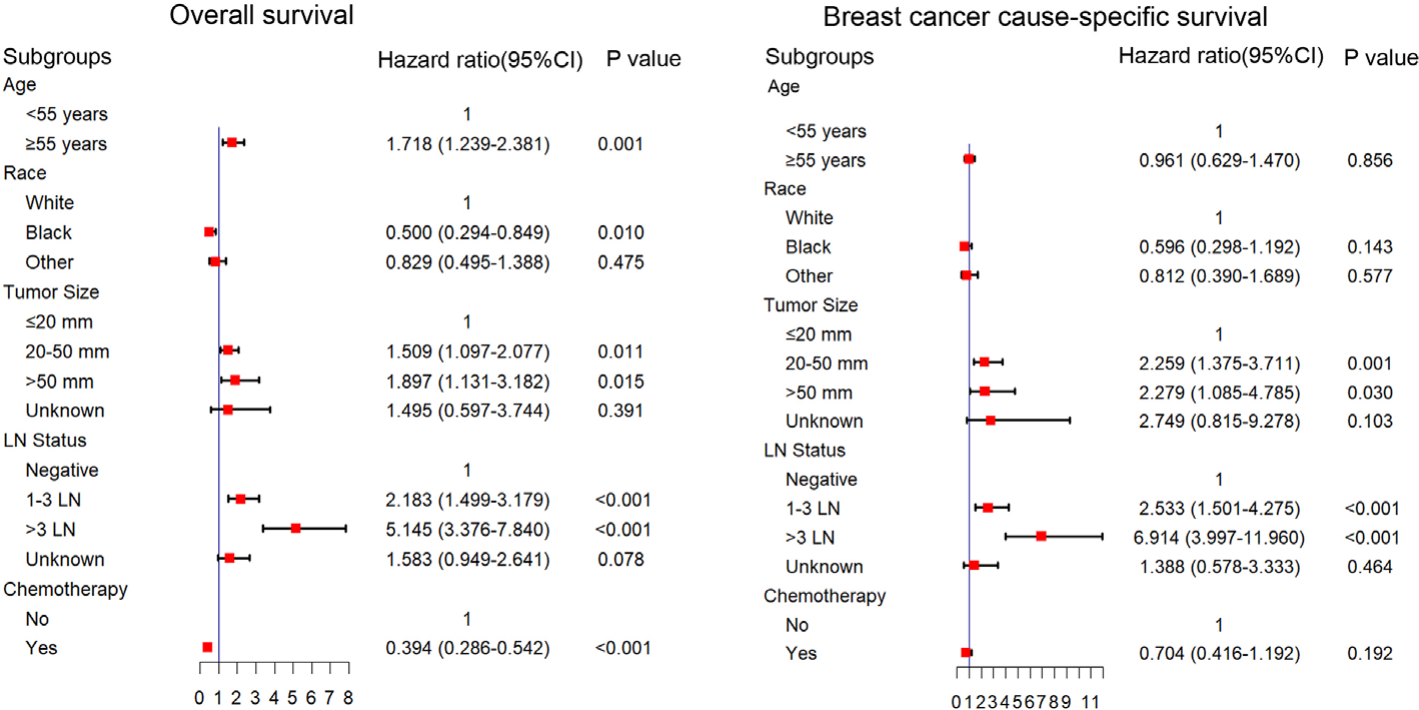
Multivariable Cox regression analysis of the factors associated with OS and BCSS in patients with a survival time longer than 5 years. OS, overall survival; BCSS, breast cancer cause-specific survival.

We explored the potential prognosis factors in long-term surviving patients with TNBC (survival time ≥5Y) using univariate and multivariable Cox regression analyses. As shown in **Table 2**, the univariate Cox regression analysis revealed that the tumor size, stage, T stage, N stage, LN status and surgery were all significantly associated with poor BCSS and OS. Other factors, including age (*p* < 0.001; ≥55y; HR = 1.899; 95% CI: 1.384–2.605), race (*p* = 0.009; black; HR = 0.492; 95% CI: 0.290–0.836), and chemotherapy (*p* < 0.001; yes; HR = 0.504; 95% CI: 0.378–0.671) were also prognostic factors for OS. The multivariable Cox regression analysis showed that age, race, tumor size, LN status, and chemotherapy were independent prognostic factors for the OS. Tumor size and LN status were independent prognostic factors for BCSS (*p* < 0.05; **Figure 1**).

**Table 2:** Prognostic factors for the overall survival (OS) and breast cancer cause-specific survival (BCSS) in patients with a survival time longer than 5 years by univariate analysis.

Parameter	OS		BCSS	
	HR (95% CI)	*p* value	HR (95% CI)	*p* value
Age				
<55 years	Reference		Reference	
≥55 years	1.899 (1.384–2.605)	<0.001	0.883 (0.584–1.335)	0.555
Race				
White	Reference		Reference	
Black	0.492 (0.290–0.836)	0.009	0.642 (0.322–1.283)	0.210
Other	0.802 (0.480–1.341)	0.400	0.872 (0.420–1.808)	0.712
Tumor size				
≤20 mm	Reference		Reference	
20–50 mm	1.397 (1.031–1.892)	0.031	2.731 (1.695–4.400)	<0.001
>50 mm	1.810 (1.109–2.953)	0.018	3.215 (1.582–6.535)	0.001
Unknown	2.033 (0.824–5.016)	0.124	3.899 (1.177–12.916)	0.026
Marital status				
Unmarried	Reference		Reference	
Married	0.927 (0.629–1.365)	0.701	0.973 (0.548–1.728)	0.927
Unknown	1.345 (0.704–2.571)	0.369	1.593 (0.643–3.947)	0.315
Laterality				
Right	Reference		Reference	
Left	0.892 (0.672–1.185)	0.432	0.840 (0.555–1.271)	0.409
Grade				
II	Reference		Reference	
III	0.816 (0.576–1.157)	0.254	0.823 (0.493–1.373)	0.455
IV	0.412 (0.057–2.993)	0.381	0.893 (0.120–6.675)	0.913
Unknown	1.319 (0.641–2.713)	0.452	2.212 (0.930–5.262)	0.073
Stage				
I	Reference		Reference	
II	1.171 (0.840–1.632)	0.352	2.442 (1.388–4.299)	0.002
III–IV	2.893 (1.988–4.210)	<0.001	7.059 (3.893–12.798)	<0.001
T stage				
T1	Reference			
T2	1.361 (0.999–1.856)	0.051	2.706 (1.661–4.410)	<0.001
T3–T4	1.994 (1.280–3.105)	0.002	3.776 (1.981–7.199)	<0.001
Unknown	1.872 (0.758–4.621)	0.174	3.659 (1.102–12.152)	0.034
N stage				
N0	Reference		Reference	
N1	1.631 (1.163–2.289)	0.005	2.760 (1.698–4.488)	<0.001
N2	3.828 (2.476–5.919)	<0.001	8.767 (5.089–15.105)	<0.001
N3	3.474 (1.816–6.644)	<0.001	3.969 (1.415–11.138)	0.009
Unknown	2.820 (0.696–11.423)	0.146	3.989 (0.547–29.078)	0.172
Metastasis				
M0	Reference		Reference	
M1	2.039 (0.652–6.380)	0.221	2.872 (0.707–11.663)	0.140
LN Status				
Negative	Reference		Reference	
1–3 LN	1.758 (1.229–2.517)	0.002	2.702 (1.627–4.489)	<0.001
>3 LN	4.435 (2.989–6.580)	<0.001	8.182 (4.862–13.770)	<0.001
Unknown	1.835 (1.110–3.033)	0.018	1.610 (0.679–3.815)	0.279
Primary site				
Upper inner quadrant	Reference		Reference	
Lower inner quadrant	1.864 (1.000–3.475)	0.050	2.280 (0.856–6.075)	0.099
Upper outer quadrant	0.991 (0.610–1.608)	0.969	1.318 (0.606–2.867)	0.487
Lower outer quadrant	1.136 (0.572–2.256)	0.715	1.682 (0.610–4.639)	0.315
Overlapping lesions	1.124 (0.666–1.895)	0.662	1.185 (0.503–2.796)	0.698
Surgery				
Breast-conserving surgery	Reference		Reference	
Mastectomy	1.429 (1.069–1.910)	0.016	1.616 (1.059–2.468)	0.026
No surgery	2.174 (1.052–4.493)	0.036	1.809 (0.558–5.871)	0.323
Radiation				
No	Reference		Reference	
Yes	0.790 (0.595–1.049)	0.103	0.956 (0.634–1.442)	0.830
Chemotherapy				
No	Reference		Reference	
Yes	0.504 (0.378–0.671)	<0.001	1.202 (0.738–1.958)	0.459

OS, overall survival; BCSS, breast cancer cause-specific survival; CI, confidence interval; LN, lymph node.

**Figure 2:**
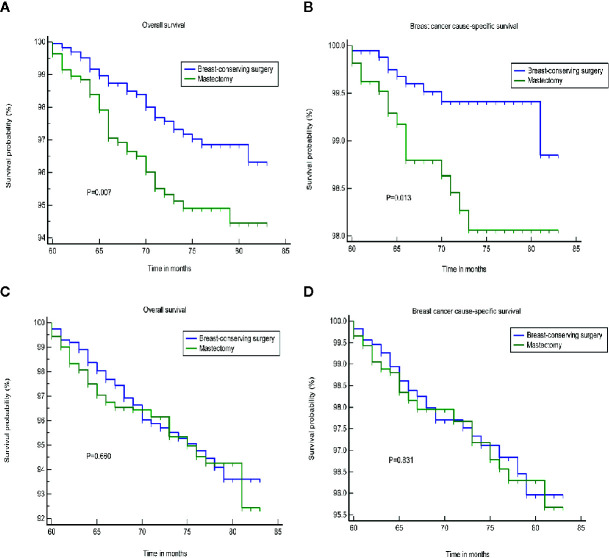
The OS and BCSS of the different treatments. **(A, B)** Tumor size less than or equal to 20 mm. **(C, D)** tumor size greater than 20 mm. OS, overall survival; BCSS, breast cancer cause-specific survival.

**Figure 3:**
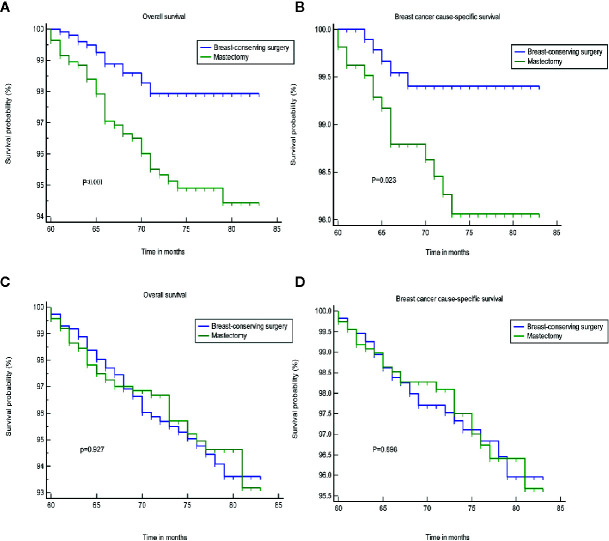
The OS and BCSS of the different treatments after PSA matching. **(A, B)** tumor size less than or equal to 20 mm. **(C, D)** tumor size greater than 20 mm. OS, overall survival; BCSS, breast cancer cause-specific survival; PSA, propensity score analysis.

We found that tumor size was a strong prognostic indicator of both OS and BCSS. A stratification analysis was performed for the groups that were treated using BCS and mastectomy based on the differences in tumor size. The results indicated that BCS resulted in a better OS and BCSS than mastectomy when the tumor size was ≤20 mm in diameter. In patients in whom the tumor size was >20 mm, the OS and BCSS were not worse following treatment with BCS than a mastectomy (**Figure 2**). A PSM was performed to reduce the bias caused by the retrospective analysis. After matching the patients who underwent BCS and a mastectomy, 2,166 patients in whom the tumor size was ≤20 mm and 2,316 patients whom the tumor size was >20 mm were included in the validation set. **Supplementary Figures S1, S2** shows the two jitter plots of the data for matched and unmatched patients, as well as the corresponding distributions of the propensity score values. The same results were obtained using a matched data set for further analysis (**Figure 3**).

**Figure 4:**
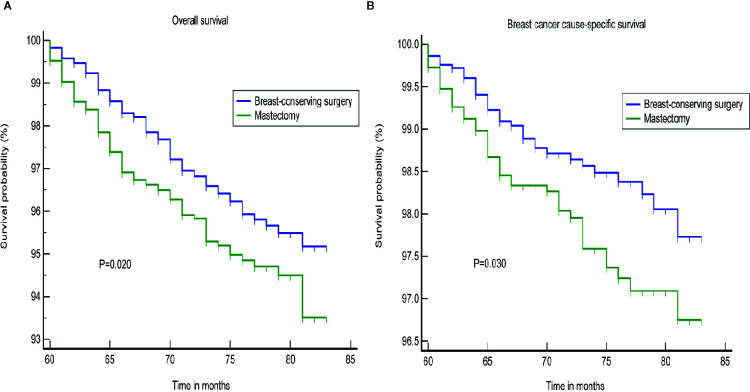
Subgroup analysis of different surgical approaches for stage I–III patients. **(A)** OS, **(B)** BCSS. OS, overall survival; BCSS, breast cancer cause-specific survival.

**Figure 5:**
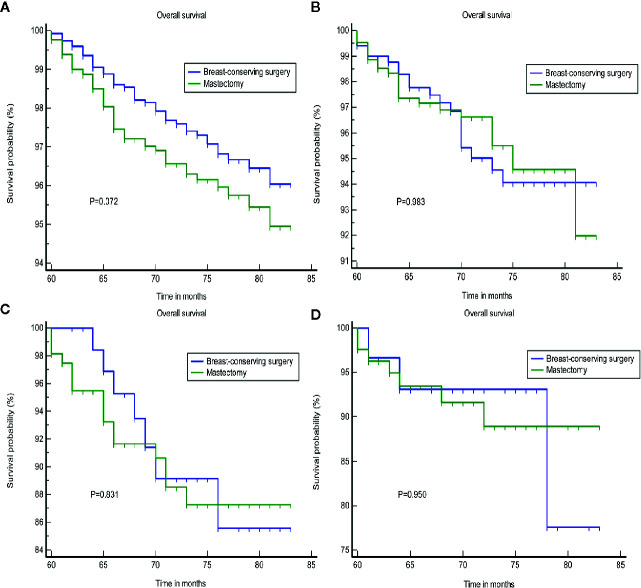
The OS of different surgical approaches stratified by LN status. **(A)** AJCC N0, **(B)** AJCC N1, **(C)** AJCC N2, and **(D)** AJCC N3. OS, overall survival; LN, lymph nodes.

Furthermore, the subgroup analyses for stage I-III patients showed superior OS and BCSS for BCS in patients with survival longer than 5 years, who were not treated with a mastectomy (*p* = 0.020; *p* = 0.030, **Figure 4**). We found that BCS, including radiotherapy, provided added benefits to the survival for patients with a tumor size less than or equal to 20 mm (**Supplementary Figure S3**). Chemotherapy may represent an important treatment option, and significantly improved the OS (**Supplementary Figure S4**). We failed to identify any difference in the groups that were treated using BCS and mastectomy according to a stratification analysis based on the LN status (**Figure 5**).

### Prognostic Nomogram for Patients Diagnosed With TNBC

**Figure 6:**
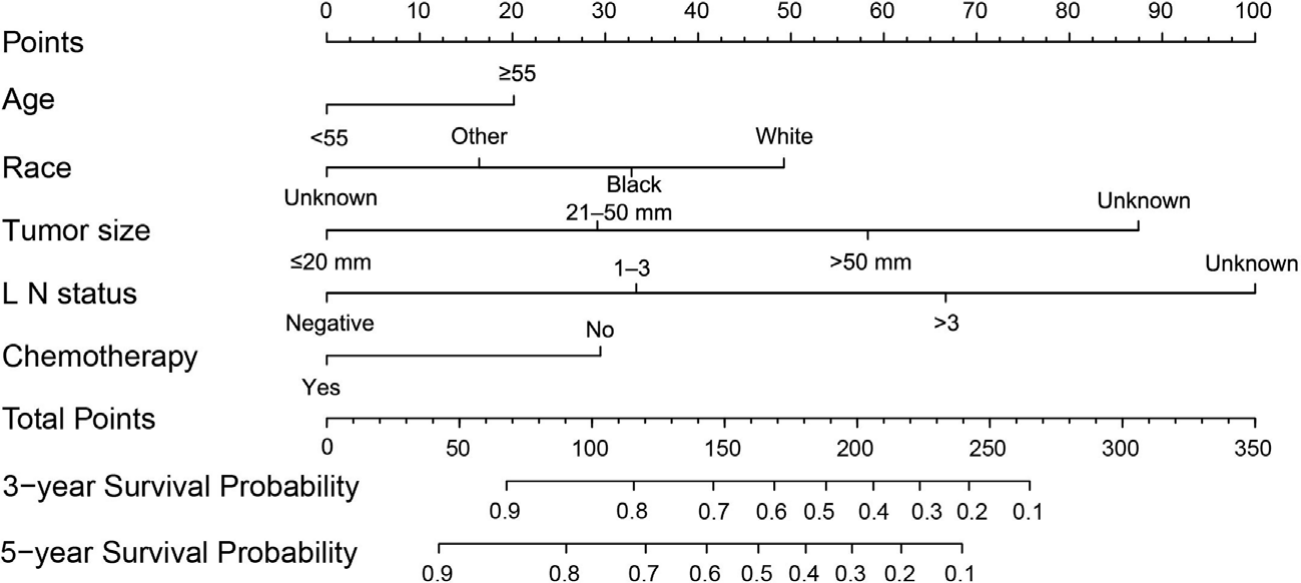
A nomogram was used to predict the 3- to 5-year survival probability for patients with TNBC. LN, lymph nodes; TNBC, triple-negative breast cancer.

A nomogram was developed based on the significant prognostic factors identified in the Cox model. In the nomogram estimation system, a weighted point value was attributed to each factor that implied a contribution to the survival prognosis. We found that TNBC patients with higher scores had a worse prognosis than that observed in those with lower scores. The final nomogram model was developed to predict the 3- and 5-year survival probability for patients diagnosed with TNBC (**Figure 6**).

**Figure 7:**
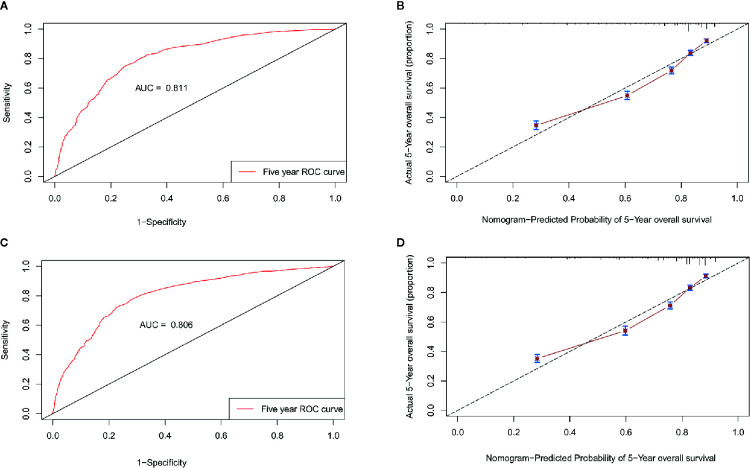
Validation of the nomogram. ROC curves and calibration plots for predicting patient survival at the 5-year time point in the training **(A, B)** and validation cohorts **(C, D)**. ROC, receiver operating characteristic curve; AUC, areas under the ROC curve.

The nomogram was validated internally in the training cohort and externally in the validation cohort. **Table S1** presents the detailed information for the validation and training cohorts, which were comparable. The predictive accuracy of the final nomogram system was determined by calculating the Harrell’s C index. The C-indexes of the nomogram in the training and validation cohorts were 0.776 (95% confidence interval [CI]: 0.767–0.785) and 0.772 (95%CI: 0.763–0.781), respectively, which were higher than the expected value of 0.7 for a system with an accurate prediction of OS. In addition, the values were higher than the C indexes for the traditional AJCC staging system in both the training and validation cohorts (0.713, 95%CI: 0.703–0.723; 0.727, 95%CI: 0.717–0.737). Moreover, the calibration plot showed that the nomogram was well calibrated (**Figure 7**). We further evaluated the effectiveness of the nomogram using the ROC curves. In the training cohort, the AUC was 0.811 and a similar AUC was observed in the validation cohort (AUC= 0.806). These findings indicate that the nomogram system constructed in this study is a better prognostic predictor for estimating the survival probability for patients diagnosed with TNBC.

## Discussion

In this study, we investigated the prognostic values of the clinicopathological characteristics and treatment outcomes in TNBC patients who survived longer than 5 years. The patients were found to exhibit distinct clinicopathological features. Our study showed that age, race, tumor size, LN status, and chemotherapy were independent prognostic factors. The results indicated that chemotherapy may represent an important treatment option. In addition, BCS provided at least an equivalent prognosis to that of a mastectomy in patients with a tumor size larger than 20 mm. Interestingly, we found that patients with a tumor size less than or equal to 20 mm may benefit from BCS rather than a mastectomy. Therefore, the type of surgery for TNBC should be considered for special subtypes among operable patients. In recent years, statistical prediction models have been developed for the majority of cancer types (7–9). For many cancers, nomograms compare favorably to traditional TNM staging systems (10, 11). We established a nomogram to predict the 3- and 5-year survival probability of TNBC patients, which was considered to be higher than that of the TNM staging systems. This represents important potential compensation for the TNM stage classification.

It appears that these aggressive clinicopathological features may be the cause of the poor outcome associated with TNBC. Compared with patients who survived longer than 5 years, patients with a survival time less than three years had higher tumor stage, T stage, N stage, distant metastasis, LN involvement, organ metastasis, and larger tumor size. LN metastasis was an independent factor associated with an unfavorable prognosis for TNBC patients. However, despite the poor prognosis of TNBC compared with other types of BC, it still remains controversial whether TNBC is prone to LN metastasis (12, 13). Our findings suggest that tumor size was a strong prognostic indicator of both OS and BCSS, which was consistent with the results of previous studies (14, 15). Most studies to date have reported a correlation between tumor size and the likelihood of LN metastasis (4, 16). We believe that with the expansion of the SEER database, more comprehensive and accurate information on prognostic factors for TNBC can be determined.

Although patients diagnosed with TNBC are highly sensitive to chemotherapy, the OS remains poor (17). Currently, the mainstay of treatment for patients diagnosed with TNBC remains cytotoxic chemotherapy (18). Our study demonstrated that the median OS of patients receiving chemotherapy was significantly different from that of patients who did not receive chemotherapy. This indicates that patients with TNBC should be given active systemic chemotherapy. Moreover, the prognoses for women with BC are continuously improving with the early detection and development of targeted therapies. Advances in targeted therapies for TNBC, include poly-ADP-ribosyl polymerase (PARP) inhibitors (19, 20), phosphoinositide 3-kinase (PI3K) pathway inhibitors (21), immune checkpoint (PD−1 and PD−L1) inhibitors (22), cyclin-dependent kinase (CDK) inhibitors (23), and a promising Trop-2 targeted antibody drug conjugate (24). To date, the study of TNBC biology and development of targeted agents is a particularly rich area of research, and patients may be suitable for treatment with more than cytotoxic chemotherapies.

Regarding surgical treatment, BCS is currently available in clinical practice. Increasing evidence indicates that TNBC might not be considered to be a contraindication for breast conservation (25, 26). Our results showed that BCS is at least equivalent to a mastectomy in terms of both the OS and BCSS. BCS offers suitable women the option to avoid undergoing a mastectomy and immediate reconstruction while promoting more rapid recovery. Moreover, greater psychosocial and self-rated satisfaction with breast appearance is achieved for BCS, regardless of the need for radiotherapy (27). Although cosmetic impairments resulting from a mastectomy can be addressed with immediate reconstruction, the benefits of improved outcomes and an avoidable deterioration in quality of life during the surgical decision-making process should still be considered. A recent study reported that for patients with long-term survival, mastectomy is associated with a poor body image, sexual health, and anxiety compared with women undergoing BCS (28). Therefore, BCS represents a preferable choice for TNBC patients if given adequate adjuvant treatment.

There are both strengths and weaknesses associated with this study. Large, well-established, and standardized populations in the SEER database were used for the analyses. However, the heterogeneous population and the retrospective nature of the data were the main limitations of the present study. Further analysis could not be carried out because of a lack of specific information related to prognoses, such as the family history, BRCA mutations, and the maternity status. The recording pattern associated with the SEER database may potentially affect the analyses. For example, while the records were not clear regarding the use of chemotherapy and radiotherapy for some patients, they may have actually received one of these treatments. Thus, these biases may underestimate the actual treatment effect. We used the PSM to solve the problem of an imbalance in the baseline characteristics between the different treatment groups. Furthermore, the application of a stratified adjusted survival analysis will improve the accuracy of the analysis. However, the two-step procedure used to estimate the causal effect is considered to be doubly robust. Therefore, additional expanded studies are required to verify our findings.

## Conclusion

The findings of the present study indicate that a localized surgical approach might be a preferable choice in TNBC patients with a survival time longer than 5 years who have distinct clinicopathological features. Age, race, tumor size, LN status, and chemotherapy were independent prognostic factors. We established a nomogram to directly quantify patient risk based on variant prognostic factors. This approach was more favorable for predicting the 3- and 5-year survival probability for patients with TNBC.

## Data Availability Statement

Publicly available datasets were analyzed in this study. This data can be found here: (https://seer.cancer.gov/).

## Author Contributions

HY and TQ contributed to the study conception and design. NX, YX, YZ, and JL contributed to the development of methodology. NX and YX contributed to acquisition of data, as well as an analysis and interpretation of data. NX and YX performed the data analysis and wrote the manuscript. HY and TQ helped to revisions of the manuscript. HY and TQ supervised the study. All authors contributed to the article and approved the submitted version.

## Funding

This study was supported by grants from the National Science and Technology Major Project (2020ZX09201021), the Medical artificial intelligence project of Sun Yat-Sen Memorial Hospital (YXRGZN201902), the National Natural Science Foundation of China (82002819, 81572596, 81972471, U1601223), the Natural Science Foundation of Guangdong Province (2017A030313828), the Guangzhou Science and Technology Major Program (201704020131), the Guangdong Science and Technology Department (2017B030314026), the Sun Yat-Sen University Clinical Research 5010 Program (2018007), and the Sun Yat-Sen Clinical Research Cultivating Program (SYS-C-201801).

## Conflict of Interest

The authors declare that the research was conducted in the absence of any commercial or financial relationships that could be construed as a potential conflict of interest.
