# Supplementary material for: Clinicopathological Characteristics and Treatment Strategies of Triple-Negative Breast Cancer Patients With a Survival Longer than 5 Years
Source: Front Oncol. 2021 Feb 1;10:617593. doi: 10.3389/fonc.2020.617593 (PMC7882729; doi:10.3389/fonc.2020.617593)
Supplement: Supplementary file 1 [file DataSheet_1.pdf]

Supplementary Material

Supplementary Figure S1 Jittered plot of the matched and unmatched observations and their distribution on propensity score values for patients with a tumor size less than 20 mm.

Supplementary Figure S2 Jittered plot of the matched and unmatched observations and their distribution on propensity score values for patients with a tumor size greater than 20 mm.

Supplementary Figure S3 Comparison of the OS for mastectomy, BCS with radiotherapy and BCS with non- radiotherapy in patients with a survival time longer than five years. **(A)** Tumor size smaller than or equal to 20 mm. **(B)** Tumor size larger than 20 mm.

Abbreviation: OS, overall survival; BCS, Breast-conserving surgery. RT, radiotherapy. NRT, non - radiotherapy.

Supplementary Figure S4 The effect of chemotherapy on OS and BCSS of TNBC patients with a survival time more than five years. **(A)** OS and **(B)** BCSS.

Abbreviation: OS, overall survival; BCSS, breast cancer cause-specific survival.

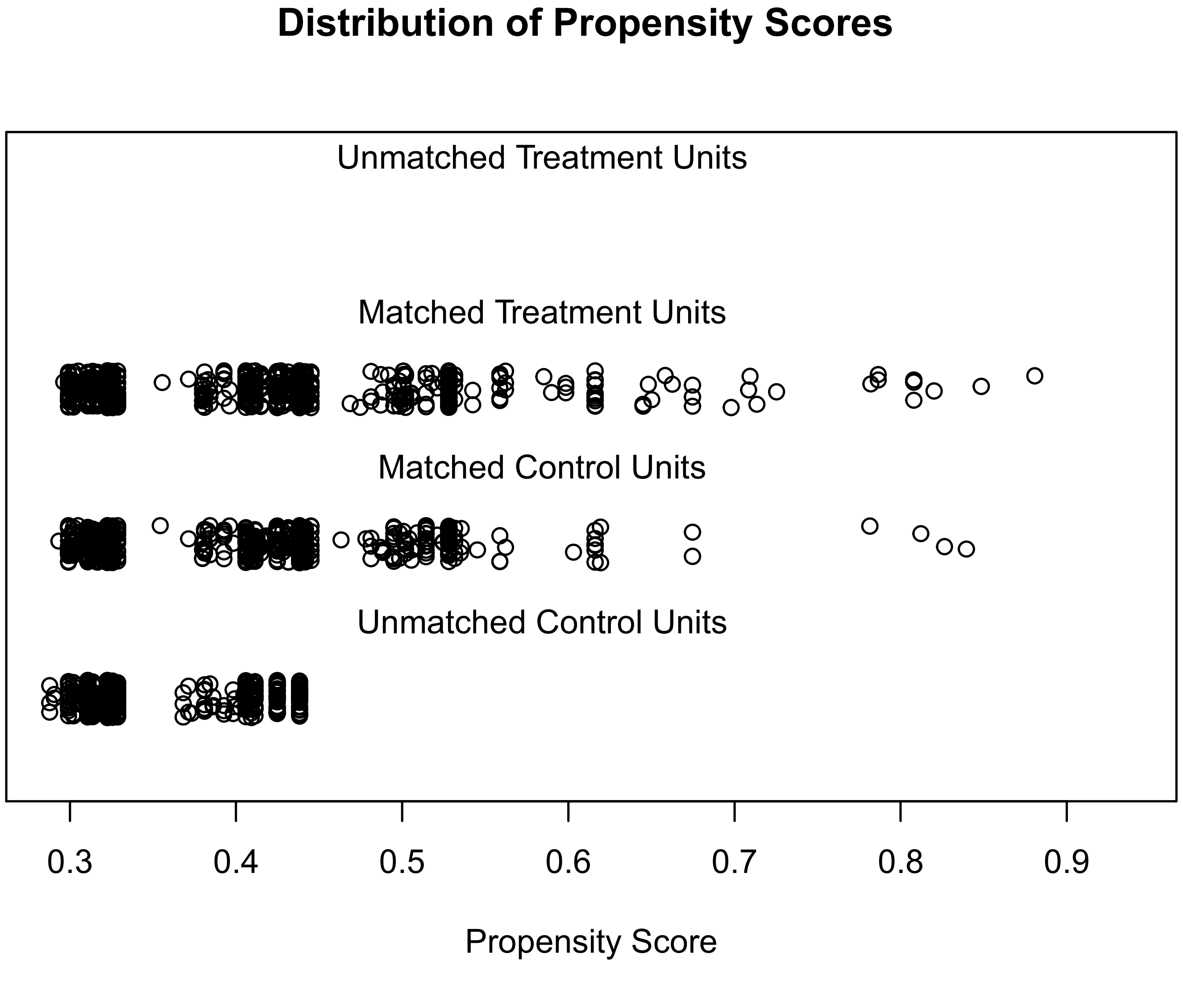

Supplementary Figure 1. Jittered plot of the matched and unmatched observations and their distribution on propensity score values for patients with a tumor size less than 20 mm.

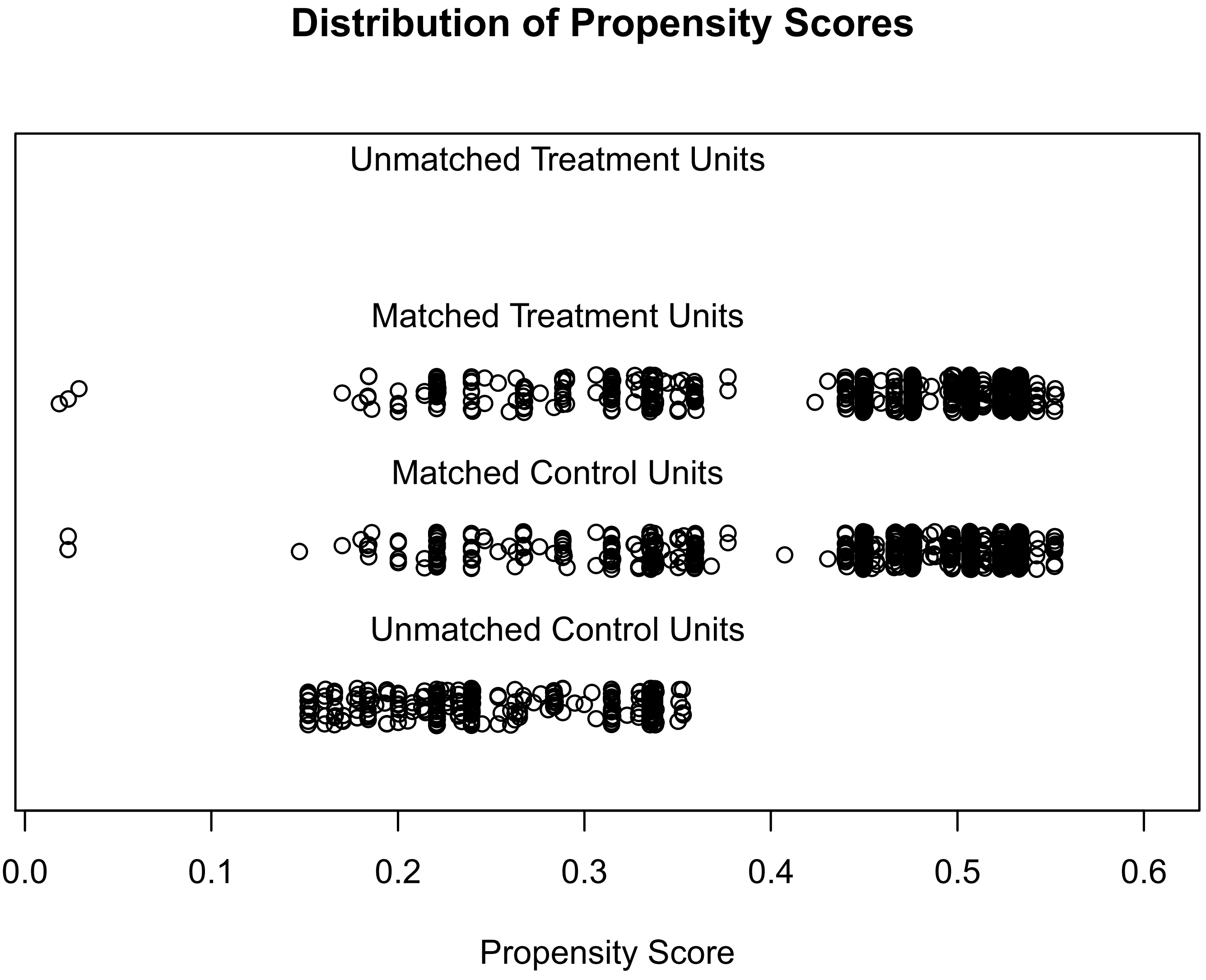

Supplementary Figure S2 Jittered plot of the matched and unmatched observations and their distribution on propensity score values for patients with a tumor size greater than 20 mm.

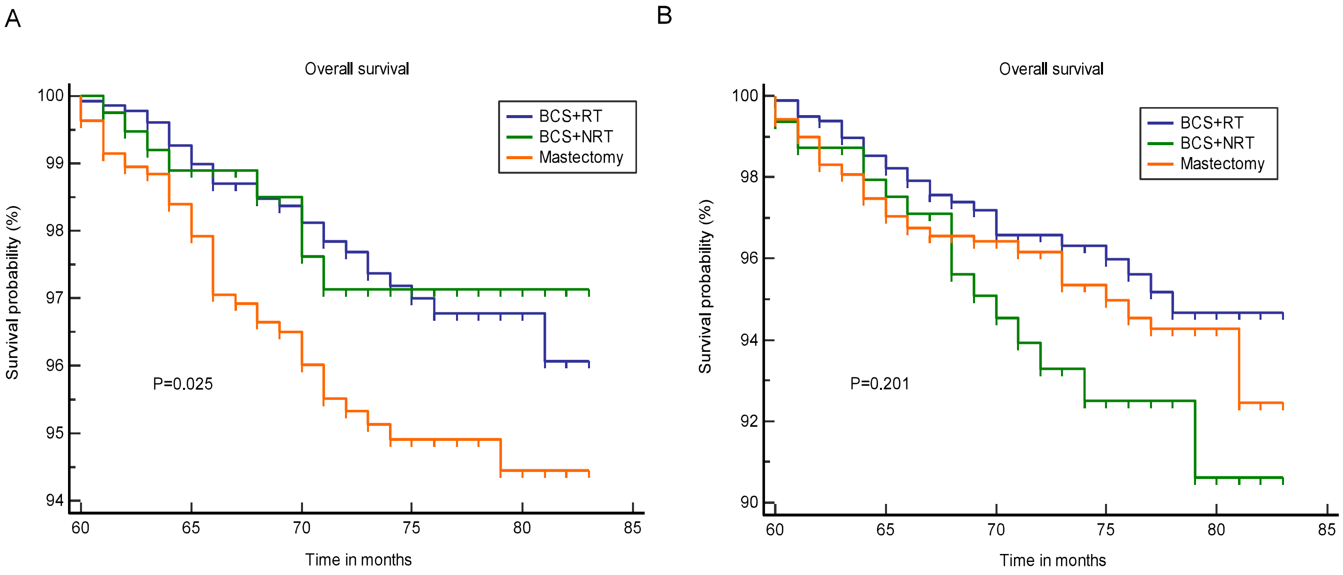

Supplementary Figure S3 Comparison of the OS for mastectomy, BCS with radiotherapy and BCS with non- radiotherapy in patients with a survival time longer than five years. **(A)** Tumor size smaller than or equal to 20 mm. **(B)** Tumor size larger than 20 mm.

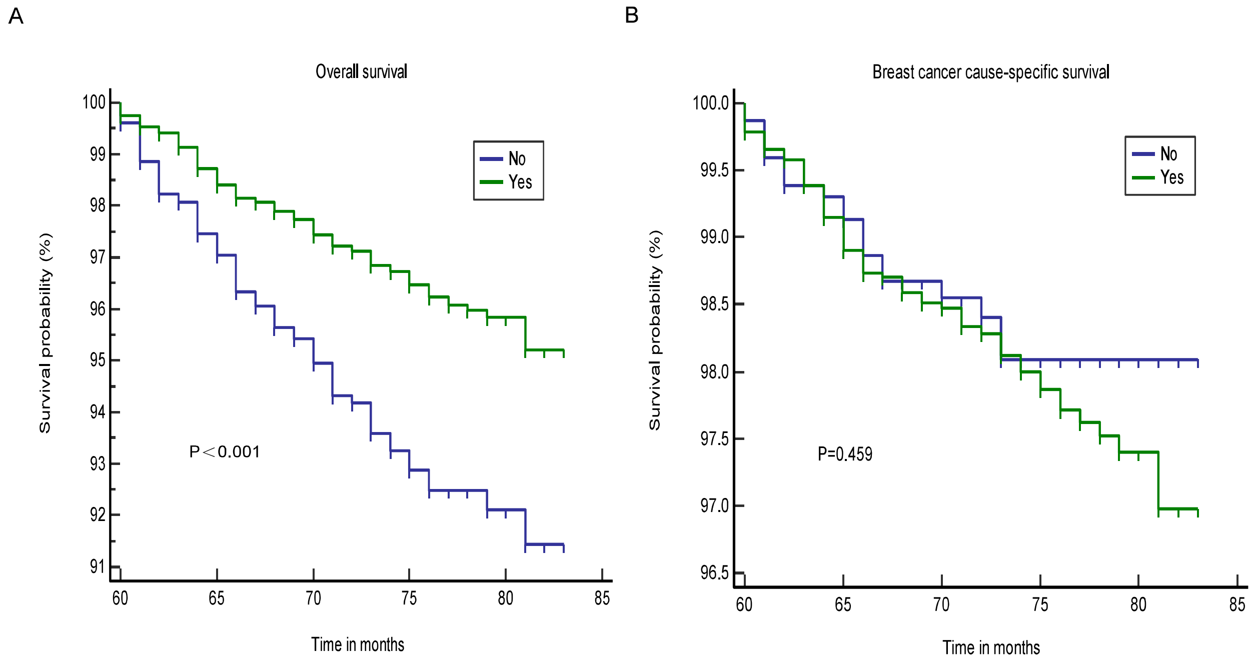

Supplementary Figure S4 The effect of chemotherapy on OS and BCSS of TNBC patients with a survival time more than five years. **(A)** OS and **(B)** BCSS.
