# Supplementary material for: Clinicopathological Characteristics and Treatment Strategies of Triple-Negative Breast Cancer Patients With a Survival Longer than 5 Years
Source: Front Oncol. 2021 Feb 1;10:617593. doi: 10.3389/fonc.2020.617593 (PMC7882729; doi:10.3389/fonc.2020.617593)
Supplement: Supplementary file 2 [file Table_1.docx]

**Table S1** Clinicopathologic characteristics of the cohort.

| **Characteristics** | | **Total cohort** | **Training cohort** | **Validation cohort** | ***p* value** |
| --- | --- | --- | --- | --- | --- |
| Age | |  |  |  | 0.543 |
|  | <55 years | 9449(37.9) | 4748(38.1) | 4701(37.7) |  |
|  | ≥55 years | 15494 (62.1) | 7724(61.9) | 7770(62.3) |  |
| Race | |  |  |  | 0.461 |
|  | White | 4404(17.7) | 2187(17.5) | 2217(17.8) |  |
|  | Black | 17788 (71.3) | 8873(71.1) | 8915 (71.5) |  |
|  | Other | 2610 (10.5) | 1343(10.8) | 1267 (10.2) |  |
|  | Unknown | 141(0.6) | 69(0.6) | 72(0.6) |  |
| Grade | |  |  |  | 0.133 |
|  | I | 549(2.2) | 272(2.2) | 277(2.2) |  |
|  | II | 4326 (17.3) | 2182 (17.5) | 2144 (17.2) |  |
|  | III | 18809(75.4) | 9423 (75.6) | 9386(75.3) |  |
|  | IV | 177 (0.7) | 94(0.8) | 83 (0.7) |  |
|  | Unknown | 1082(4.3) | 501(4.0) | 581 (4.7) |  |
| Tumor size | |  |  |  | 0.664 |
|  | ≤20mm | 9440(37.8) | 4681(37.5) | 4759(38.2) |  |
|  | 21-50mm | 8943(35.9) | 4472(35.9) | 4471(35.9) |  |
|  | >50mm | 2569(10.3) | 1298(10.4) | 1271(10.2) |  |
|  | Unknown | 3991(16.0) | 2021(16.2) | 1970(15.8) |  |
| Marital status | |  |  |  | 0.472 |
|  | Unmarried | 4322(17.3) | 2181(17.5) | 2141(17.2) |  |
|  | Married | 19276 (77.3) | 9638 (77.3) | 9638 (77.3) |  |
|  | Unknown | 1345 (5.4) | 653(5.2) | 692(5.5) |  |
| Laterality | |  |  |  | 0.394 |
|  | Right | 12094 (48.5) | 6048 (48.5) | 6046(48.5) |  |
|  | Left | 12795 (51.3) | 6392 (51.3) | 6403 (51.3) |  |
|  | Others | 54 (0.2) | 32(0.3) | 22(0.2) |  |
| Stage | |  |  |  | 0.850 |
|  | 0 | 2(0.0) | 1(0.0) | 1(0.0) |  |
|  | I | 7791 (31.2) | 3872(31.0) | 3919(31.4) |  |
|  | II | 9035 (36.2) | 4541(36.4) | 4494 (36.0) |  |
|  | III | 3113(12.5) | 1547(12.4) | 1566(12.6) |  |
|  | IV | 1354(5.4) | 662(5.3) | 692(5.5) |  |
|  | Unknown | 3648(14.6) | 1849(14.8) | 1799(14.4) |  |
| T stage | |  |  |  | 0.784 |
|  | 0 | 87(0.3) | 46(0.4) | 41(0.3) |  |
|  | T1 | 9293(37.3) | 4607(36.9) | 4686(37.6) |  |
|  | T2 | 8498 (34.1) | 4252(34.1) | 4246 (34.0) |  |
|  | T3-T4 | 3321 (13.3) | 1670(13.4) | 1651 (13.2) |  |
|  | Unknown | 3744 (15.0) | 1897 (15.2) | 1847(14.8) |  |
| N stage | |  |  |  | 0.401 |
|  | N0 | 13974(56.0) | 6952(55.7) | 7022(56.3) |  |
|  | N1 | 5175 (20.7) | 2631(21.1) | 2544 (20.4) |  |
|  | N2 | 1255 (5.0) | 615(4.9) | 640 (5.1) |  |
|  | N3 | 1045 (4.2) | 505(4.0) | 540 (4.3) |  |
|  | Unknown | 3494(14.0) | 1769(14.2) | 1725(13.8) |  |
| Metastasis | |  |  |  | 0.413 |
|  | M0 | 20445(82.0) | 10209(81.9) | 10236(82.1) |  |
|  | M1 | 1354(5.4) | 662(5.3) | 692(5.5) |  |
|  | Unknown | 3144(12.6) | 1601(12.8) | 1543(12.4) |  |
| LN Status | |  |  |  | 0.654 |
|  | Negative | 14588(58.5) | 7248(58.1) | 7340(58.9) |  |
|  | 1-3 LN | 4167 (16.7) | 2092(16.8) | 2075 (16.6) |  |
|  | >3 LN | 1900 (7.6) | 958(7.7) | 942 (7.6) |  |
|  | Unknown | 4288 (17.2) | 2174(17.4) | 2114 (17.0) |  |
| Surgery | |  |  |  | 0.638 |
|  | No surgery | 2562 (10.3) | 1270 (10.2) | 1292 (10.4) |  |
|  | Breast-conserving surgery | 11546 (46.3) | 5816 (46.6) | 5730 (45.9) |  |
|  | Mastectomy | 10790(43.3) | 5366(43.0) | 5424 (43.5) |  |
|  | Unknown | 45 (0.2) | 20 (0.2) | 25(0.2) |  |
| Radiation | |  |  |  | 0.260 |
|  | No | 13560(54.4) | 6736(54.0) | 6824(54.7) |  |
|  | Yes | 11383 (45.6) | 5736(46.0) | 5647 (45.3) |  |
| Chemotherapy | |  |  |  | 0.810 |
|  | No | 7347(29.5) | 3665(29.4) | 3682(29.5) |  |
|  | Yes | 17596 (70.5) | 8807(70.6) | 8789 (70.5) |  |
| Bone metastasis | |  |  |  | 0.289 |
|  | No | 24276(97.3) | 12152(97.4) | 12124(97.2) |  |
|  | Yes | 667(2.7) | 320(2.6) | 347(2.8) |  |
| Brain metastasis | |  |  |  | 0.369 |
|  | No | 24763(99.3) | 12388(99.3) | 12375(99.2) |  |
|  | Yes | 180(0.7) | 84(0.7) | 96(0.8) |  |
| Liver metastasis | |  |  |  | 0.065 |
|  | No | 24511(98.3) | 12237(98.1) | 12274(98.4) |  |
|  | Yes | 432(1.7) | 235(1.9) | 197(1.6) |  |
| Lung metastasis | |  |  |  | 0.968 |
|  | No | 24322(97.5) | 12161(97.5) | 12161(97.5) |  |
|  | Yes | 621(2.5) | 311(2.5) | 310(2.5) |  |
| Status | |  |  |  | 0.936 |
|  | Alive | 19542(78.3) | 9774(78.4) | 9768(78.3) |  |
|  | Dead | 5401(21.7) | 2698(21.6) | 2703(21.7) |  |
| First malignant primary indicator | | |  |  | 0.320 |
|  | No | 20077(80.5) | 10070(80.7) | 10007(80.2) |  |
|  | Yes | 4866(19.5) | 2402(19.3) | 2464(19.8) |  |

**Abbreviations:** LN, lymph node.
